# Hybrid e-rehabilitation services: SMART-system for remote support of rehabilitation activities and services

**DOI:** 10.5195/ijt.2022.6480

**Published:** 2022-05-20

**Authors:** Oleksandr V. Palagin, Kyrylo S. Malakhov, Vitalii Yu. Velychko, Tetiana V. Semykopna

**Affiliations:** V. M. Glushkov Institute of Cybernetics of the National Academy of Sciences of Ukraine

**Keywords:** Digital library of media content, Distributional semantics, Hybrid e-rehabilitation medicine, Ontology engineering, Rehabilitation, Telerehabilitation, Transdisciplinary intelligent information and analytical system for the rehabilitation processes support in a pandemic (TISP), UkrVectōrēs, vHealth

## Abstract

One of the most effective solutions in medical rehabilitation assistance is remote patient / person-centered rehabilitation. Rehabilitation also needs effective methods for the “Physical therapist – Patient – Multidisciplinary team” system, including the statistical processing of large volumes of data. Therefore, along with the traditional means of rehabilitation, as part of the “Transdisciplinary intelligent information and analytical system for the rehabilitation processes support in a pandemic (TISP)” in this paper, we introduce and define: the basic concepts of the new hybrid e-rehabilitation notion and its fundamental foundations; the formalization concept of the new Smart-system for remote support of rehabilitation activities and services; and the methodological foundations for the use of services (UkrVectōrēs and vHealth) of the remote Patient / Person-centered Smart-system. The software implementation of the services of the Smart-system has been developed.

The methodology of rehabilitation measures in a pandemic has several significant features associated with the unpredictability and high rate of emergence of problems of high complexity, limited communication between the therapist and the patient, the need for high responsiveness of decision-making and their compliance, the scale of the process and the associated need to use scalable operating tools, etc. One of the most effective solutions in medical rehabilitation assistance is remote patient/personal-centered rehabilitation. It requires online monitoring tools, telemetry and interventions focused on the patient's capabilities, developed Internet interaction, intelligent information technologies, and services. Patient / Person-centered rehabilitation also needs effective methods in the “Physical therapist – Patient – Multidisciplinary team” system, statistical processing of large volumes of data, etc. Therefore, along with the traditional means of rehabilitation, as part of the “Transdisciplinary intelligent information and analytical system for the rehabilitation processes support in a pandemic (TISP)” (Palagin, 2021) the Smart-system for remote support of rehabilitation activities and services (Smart-system) was developed. Combined with intelligent remote biofeedback (Palagin & Kurgaev, 2021) devices and effective miniature remote monitoring, telemetry, and recovery devices (embedded systems and wearable devices) (Palagin et al., 2020a), such systems hold great promise, as evidenced by worldwide experience as well. That research would not have been possible without the financial support of the National Research Foundation of Ukraine (NRFU) (NRFU, n.d.). The project “Transdisciplinary intelligent information and analytical system for the rehabilitation processes support in a pandemic (TISP)” won the competition “Science for Human and Social Security” and received grant funding in 2020.

The objective of the research described herein is to develop a formal model, software implementation, and the methodological foundations for the use of services of the remote Patient / Person-centered Smart-system for providing medical rehabilitation assistance to patients in a pandemic (i.e., the novel coronavirus disease COVID-19).

Smart-system for remote support of rehabilitation activities and services is a complex, integrated, patient / person-centered information subsystem of TISP for the provision of medical care, solving various clinical, organizational, and research tasks in the field of rehabilitation medicine: consultations; remote observation and support of rehabilitation processes and activities; classification, forecasting, and knowledge extraction; research and review of the new domain areas; use of remote communication technologies; elements of artificial intelligence, in particular, ontology engineering; (Palagin et al., 2011a; Palagin et al., 2011b; Palagin et al., 2014; Palagin et al., 2018; Velychko et al., 2014) and machine learning (Palagin et al., 2020b).

According to the World Health Organization, rehabilitation is a set of processes designed to ensure that people with impaired functions due to illness, injury, and birth defects, adapt to new living conditions in the society in which they live. Rehabilitation of people who have recovered from COVID-19 disease is, of course, an extremely important social problem. This fact is already well understood by the international medical community. At the same time, the availability of rehabilitation measures in different countries is quite different. In countries with developed insurance medicine, the rehabilitation process in the corresponding specialized centers is available to many. Unfortunately, in Ukraine the situation is different. The needs of patients for outpatient rehabilitation services far exceed the available resources; this requires finding alternative solutions and connecting modern and advanced technologies to support patients. Therefore, there is a great need for the use of a new modern direction of rehabilitation medicine: telerehabilitation.

In the modern world, the generally accepted definition of the concept of telerehabilitation or E-rehabilitation (Palagin et al., 2020a) is a complex of rehabilitation exercises and training programs that are provided to the patient remotely using telecommunication computer technologies, mainly at the outpatient stage of treatment. The meaning of this modern trend is that the patient independently, usually at home, performs rehabilitation programs under the remote control and guidance of a specialist. Telerehabilitation should be accompanied by appropriate software that allows the rehabilitation specialist who observes the patient in the hospital to quickly compile an individual set of exercises for self-study in video format. This complex should be adjusted depending on the dynamics of recovery, which is monitored using miniature tools for the functional assessment at home. Thus, patients have the opportunity to continue the structured home rehabilitation program developed in the inpatient phase.

Telerehabilitation is the embodiment of several modern technological trends at once. First of all, telerehabilitation is impossible without telecommunication technologies. Secondly, telerehabilitation requires the usage of specialized hospital information systems (HIS, also known as hospital management software or hospital management systems) for rehabilitation. These systems are needed both for the administration of patients and (this is the most important part), for the creation of a number of rehabilitation documents in accordance with the structure of the rehabilitation cycle, for example, an individual rehabilitation plan, categorical profile of a patient, rehabilitation prognosis, etc. Thirdly, an important part of telerehabilitation technologies is the functional assessment of the patient's condition at home using miniature devices (embedded systems and wearable devices) and modern algorithms for evaluating data in accordance with the point-of-care testing trend, which is carried out directly at the patient's location, outside the doctor's office. Finally, telerehabilitation is impossible without the active involvement of the patient in the decision-making process for their diagnosis and treatment. This is one of the main trends in modern medicine, which was supported, among other things, at the level of legislative initiatives in the health care system of Ukraine.

## BASIC CONCEPTS OF THE HYBRID E-REHABILITATION NOTION

**Figure 1:**
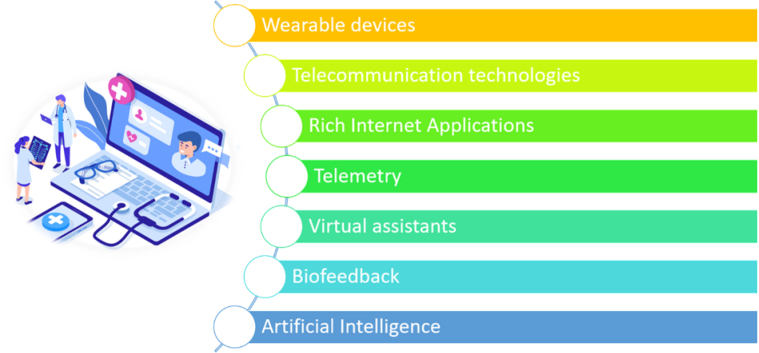
Fundamental Methods, Approaches and Technologies of the Hybrid E-rehabilitation Notion

The rapid development of telerehabilitation worldwide and the acquisition by this direction of medicine of transdisciplinary connections with various subject areas that go beyond the modern paradigm of e-health, led to the emergence of the most modern type of rehabilitation medicine – Hybrid e-rehabilitation medicine. The Hybrid e-rehabilitation notion (shown in Figure 1) consists of a number of the following fundamental methods, approaches, and technologies:

- *Telecommunication technologies*. This means the delivery of rehabilitation services over telecommunication networks and the internet. Also, this allows patients to interact with providers remotely and can be used both to assess patients and to deliver therapy.
- *Rich Internet Applications (RIA)*. These are chiefly the Hospital Information Systems (HIS). A HIS is a comprehensive, integrated information system designed to manage all aspects of a hospital's operation, such as medical, administrative, financial, and legal issues and the corresponding processing of services. Another feature of such systems is the provision of communication between patients and doctors from the multidisciplinary rehabilitation team.
- *Telemetry*. This is a set of technologies that allow remote measurement, and collection and transmission of information about performance indicators (physiological parameters) of the patient's body in real-time.
- *Wearable devices and Embedded systems*. Wearables may be for widespread use in which case they are just a particularly small example of mobile computing. Alternatively, they may be for special purposes such as fitness trackers or medical devices. They may incorporate special sensors such as accelerometers, heart rate monitors, or on the more advanced side, electrocardiogram (ECG), blood oxygen saturation (SpO2) monitors and blood pressure monitoring devices. These functions are often bundled together in a single unit, like an activity tracker or a smartwatch like the Apple Watch Series or Samsung Galaxy Gear Sport. Devices such as these are used for physical training and monitoring overall physical health, as well as for alerting to serious medical conditions such as seizures (e.g., Empatica Embrace). Currently, other applications within healthcare are being explored, such as: forecasting changes in mood, stress, and health; measuring blood alcohol content; measuring athletic performance; long-term monitoring of patients with heart and circulatory problems that records an electrocardiogram and is self-monitoring; health risk assessment applications, including measures of frailty and risks of age-dependent diseases; and automatic documentation of care activities.
- *Biofeedback (BF)*. Biofeedback is the process of gaining greater awareness of many physiological functions of one's own body. Biofeedback (Palagin & Kurgaev, 2021) may be used to improve health, performance, and the physiological changes that often occur in conjunction with changes to thoughts, emotions, and behavior. Recently, technologies have provided assistance with intentional biofeedback. Humans conduct BF naturally all the time, at varied levels of consciousness and intentionality. BF and the biofeedback loop can also be thought of as self-regulation. Some of the processes that can be controlled include brainwaves, muscle tone, skin conductance, heart rate, and pain perception. The essence of BF-method from a technical point of view is computer registration in real-time of certain physiological parameters that are not available for direct human perception (EEG, electrical resistance of the skin, the heart rate, body temperature, etc.) and their transformation into a form natural to humans. The method is based on the principle of translating information in the form of electrical physiological signals received from a human body using special sensors into feedback information in the form of images, natural messages, multimedia, games, and other forms of information and material interaction in a given range of values. BF, along with other well-known methods, is included in the list of treatments officially used in medical rehabilitation in Europe, and in the United States alone, BF-methods are implemented in more than 700 clinical centers.
- *Intelligent virtual / personal assistants*. These assistants are the software agents that can perform tasks or services for an individual based on commands or questions. Virtual assistants use natural language processing to match user text or voice input to executable commands. Many such assistants continually learn using artificial intelligence techniques including machine learning and computational linguistics with distributional semantic modeling in vector spaces (Palagin et al., 2020b). Modern software agents of the class of intelligent virtual assistants can interact with each other to perform a certain class of tasks. The term “chatbot” is sometimes used to refer to virtual assistants generally or specifically accessed by online chat. Some virtual assistants are able to interpret human speech and respond via synthesized voices. In particular, within the framework of the project and the TISP system (Litvin, et al., 2021), a universal dialogue subsystem (UDS) was developed and implemented within the Physical Medicine and Rehabilitation (PM&R) domain area in the form of a web application and a virtual interlocutor in the “Telegram” service. The developed UDS of TISP system uses the ontology-related approach, in particular, the ontology representations of the White Book on Physical and Rehabilitation Medicine in Europe (WB; Negrini, 2018) WB and the International Classification of Functioning, Disability and Health (ICF; ICF, n.d.). The UDS (Litvin, 2021) is based on the new technique which assumes the presence of several query templates with each corresponding to a special semantic type of question. Meaningful entities extracted from the user's natural language phrase are substituted into the corresponding query template. For the most suitable query template selection a tree based semantic analysis method is proposed. The method assumes that the frame is shifting through the words list of the phrase, considering on each step one or several words. These words are analyzed to match one of the conditions on the current tree position. The most matching condition determines the following position on the next level of the tree. The process proceeds until there will remain the only option of query template and all the sufficient conditions for the corresponding semantic type are proved to be observed. Then depending on the selected query template input entities for it are taken from the given positions of the previous consideration.
- *Artificial Intelligence methods and applications for big data processing to knowledge extraction and solving analytical tasks* (Gladun, et al., 2008). For Knowledge Discovery and solving the main analytical tasks, such as classification, diagnostics, or prediction we use the method called Growing Pyramidal Network (GPN; Gladun, et al., 2008). GPN inductive training is performed on the basis of precedents sets. The result of training is a regularity in the form of a logical function. GPN belongs to the class of statistical methods. Intelligence information search is based on predictive models of distributional semantics. The GPN method and GPN based software has some unique features. These are: search for all possible combinations of values of objects attributes for allocating the most important combinations of attributes' values for building the model of classes of objects; guaranteed finding the most significant combinations of attributes values using the principle of minimal length of the hypothesis description (logical expression); always 100% correct classification of all objects from training set; works with data of any complexity and possibility to discover a regularity however complex it be; and automatic clustering of objects set. In a pyramidal network, the complexity of decision functions is automatically adjusted to the data from the training set, depending on the compactness of the training set. There is no need for a test set to verify the quality of the found logical regularities. In the process of using regularities, the amount of information contained in the logical regularities is counted. If it has changed, then a decision to retrain the system is made. There is high speed recognition of new objects (i.e., constant, not depending on the volume of stored information). If no exact solution found – all possible solutions (Including the decision “I do not know”) is presented and ranked according to the confidence level for each. There is an available explanation mode in which there are explained grounds of the decision for each of the recognizable objects. GPN methods powered the clinical decision support subsystem of TISP.

The typical representative of systems that implement the concept of the hybrid E-rehabilitation is the TISP system and, in particular, its patient/person-centered information subsystem, a Smart-system for remote support of rehabilitation activities and services. Consider the developed general formalization of the Smart-system.

## FORMALIZATION CONCEPT OF SMART-SYSTEM FOR REMOTE SUPPORT OF REHABILITATION ACTIVITIES AND SERVICES

The generalized formalization concept of the Smart-system for remote support of rehabilitation activities and services is represented as a three-tuple *S* using the revised formalism given in (Palagin et al., 2018):


(1) S=〈D,F,E〉

Where:

*S* – is the Smart-system for remote support of rehabilitation activities and services (subsystem of TISP).

*D* – is a set of web services (RIA) and desktop applications that are available for usage in the Smart-system *S* :


(2) $$D=\overset{k}{\underset{j=1}{\Sigma }}Z_{T_{j}}$$

Where:


$$Z_{T_{j}}$$

is a web service or desktop application that implements specific algorithms, processes, and functions of the Smart-system *S* (*j* = 1,*k*, *k* ∈ ℕ, *k* – the number of developed web services and desktop applications that are part of the Smart-system *S*).


$$F=S:{\left\{C_{i}|i=\overset{—}{1,n}\right\}}_{n\in \mathbb{N}}$$

is a set of functions, the functional filling-up of the Smart-system *S*, each function is the result of coordination and interaction of the Smart-system *S* elements.


$$C_{i}\subseteq D,C_{i}={\left\{D_{y}|y\geq 1,y\leq m\right\}}_{y\in \mathbb{N},m\in \mathbb{N}}$$

is a subset of web services and desktop applications that are required to implement the *j*-th function of Smart-system *S*. The formation of this subset allows creating personalized pipelines and scenarios for using the developed web services and desktop applications (as well as the use of additional external software), which enables more flexibly in use of the services of the Smart-system for PM&R doctors. The formation of such pipelines and scenarios is beyond the scope of this article and should be considered separately.

At the current stage of development, the Smart-system includes various technologies, web services, and desktop applications, in particular:


(3) $$\overset{6}{\underset{j=1}{\Sigma }}Z_{T_{i}}=\left\{T_{1},T_{2},T_{3},,T_{4},T_{5},T_{6}\right\}$$

Where:

*T*_1_ – *Remote consulting service* – Smart-system's telemedicine module (which provides online video and audio communication) using modified open-source software Jitsi Meet (JitsiMeet, n.d.).

*T*_2_ – *Digital doctor's office* of specialized medical care service, in particular the PM&R doctors. In the personal digital office, there are services for managing online appointments, including video consultations, personal doctor's and patient's profiles, fiscal management, a calendar for scheduling and planning consultations, access to the electronic health records (i.e., patient's profiles and health history), and a module for carrying out electronic advisory conclusions on the results of the video consultation.

*T*_3_ – *Service for automated processing and integration of all basic workflows* of a medical institution for interaction between an administrator, a doctor, and a patient.

*T*_4_ – *Collaborative service* for the creation and use of file-sharing and exchange services, in particular, for interacting and exchanging medical digital images via Digital Imaging and Communications in Medicine (DICOM). DICOM is the standard for the communication and management of medical imaging information and related data. DICOM is most commonly used for storing and transmitting medical images enabling the integration of medical imaging devices such as scanners, servers, workstations, printers, network hardware, and picture archiving and communication systems (PACS) from multiple manufacturers. It has been widely adopted by hospitals and is making inroads into smaller applications such as dentists' and doctors' offices. DICOM incorporates standards for imaging modalities such as radiography, ultrasonography, computed tomography, magnetic resonance imaging, and radiation therapy. DICOM includes protocols for image exchange (e.g., via portable media such as SD cards), image compression, 3-D visualization, image presentation, and results reporting.

*T*_5_ – *UkrVectōrēs* (UkrVectōrēs, n.d.) web service. This is a NLU-powered toolkit for knowledge discovery, classification, diagnostics, and prediction – an entities similarity tool. You can think about UkrVectōrēs as a kind of “cognitive-semantic calculator.” The online toolkit UkrVectōrēs covers the following elements of distributional analysis (Palagin et al., 2020b): calculates semantic similarity between pairs of words; finds words semantically closest to the query word; applies simple algebraic operations to word vectors (addition, subtraction, finding average vector for a group of words and distances to this average value); draws semantic maps of relations between input words (it is useful to explore clusters and oppositions, or to test your hypotheses about them); gets the raw vectors (arrays of real values) and their visualizations for words in the chosen model; downloads default models; and uses other prognostic models distributive semantics freely distributed, by adjusting the configuration file.

*T*_6_ – *vHealth Electronic Library service* (vHealth, n.d.). This is a distributed information system that allows you to store, use and share various collections of electronic documents (video and audio content) of arbitrary domain areas for distance learning of patients and their relatives, in particular, a rehabilitation complex of exercises and activities.

Consider in detail some services and applications developed according to the generalized formalization concept of the Smart-system for telemedicine support of rehabilitation measures, in particular, UkrVectōrēs and vHealth services.

## UKRVECTŌRĒS – AN NLU-POWERED TOOL FOR KNOWLEDGE DISCOVERY, CLASSIFICATION, DIAGNOSTICS, AND PREDICTION

The distributed numerical feature representations of words (word embeddings) and word vector space models, as a result, are well established in the field of computational linguistics and have been here for decades, see Turney & Pantel (2010) and Palagin et al., (2020b) for an extensive review. However, recently they received substantially growing attention. Learning word representations lies at the very foundation of many natural language processing (NLP) tasks because many NLP tasks rely on good feature representations for words that preserve their semantics as well as their context in a language.

The network service UkrVectōrēs computes the semantic relations (similarity) between the entities of the Ukrainian language within the selected distributional semantic model of the vector representation of entities (entities embeddings). UkrVectōrēs is a natural language distributional analysis and distributional semantic modeling web service (toolkit), a natural language research technique based on the study of the environment (distribution), individual entities in the text without the full lexical or grammatical meanings of these entities. In the general case, distributional analysis, and distributional semantic modeling (Palagin et al., 2020) use, base, and examine the essence of a natural language, such as words or phrases. Within the framework of this method, an ordered set of universal procedures is applied to texts in natural language, which makes it possible to single out the basic units of the language (phonemes, morphemes, words, phrases), to classify them, and to learn the relation of semantic similarity between them.

The network service UkrVectōrēs is a tool that allows exploring the semantic relationships between entities in the framework of predictive models of distributional semantics (PMDS), using an open-source software library genism (Genism, n.d.) for processing and mathematical modeling of the natural language (including an application programming interface for different algorithms such as Word2vec, fastText, etc.). The user can choose one or several carefully prepared predictive models of distributional semantics (or use other models of vector representation for words of the Ukrainian language) learned on various text corpora, in particular, such as the WB (Negrini, 2018) dataset.

The UkrVectōrēs service covers the following elements of the distributional semantic analysis / modeling:

- computation of semantic similarity between pairs of entities (words) within the selected PMDS;
- computation of the entity closest to a given one within the selected PMDS (computation of semantic associates). In distributional semantics, words are usually represented as vectors in a multi-dimensional space of their contexts. Semantic similarity between two entities is then calculated as a cosine similarity between their corresponding vectors; it takes values between *-1* and *1* (usually only values above *0* are used in practical tasks). *0* value roughly means the entities lack similar contexts, and thus their meanings are unrelated to each other. *1* value means that the entities' contexts are absolutely identical, and thus their meaning is quite similar;
- applying simple algebraic operations to entity vectors (addition, subtraction, finding average vector for a group of entities and distances to this average value) within the selected PMDS;
- generation of the semantic maps (using the open-source software toolkit TensorFlow) of relations between input entities (it is useful to explore clusters and oppositions, or to test your hypotheses about them);
- using other freely shared PMDS via special configuration file.

*Let us consider the methodology of a user experience with the graphical user interface of the UkrVectōrēs single-page application*, in particular, for the distributional semantic analysis of natural language texts.

**Figure 2:**
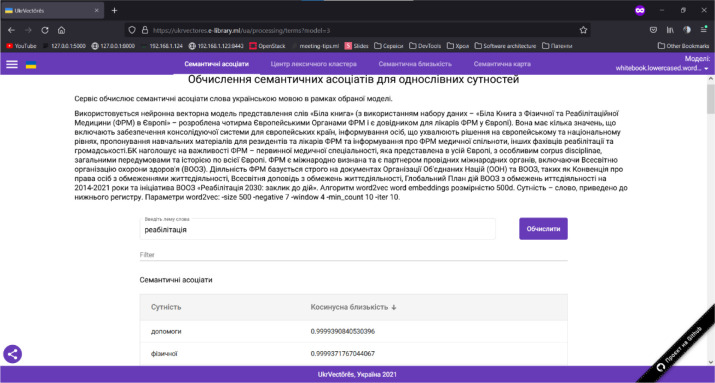
GUI of the UkrVectōrēs SPA Service (“Semantic Associates” Mode)

**Figure 3:**
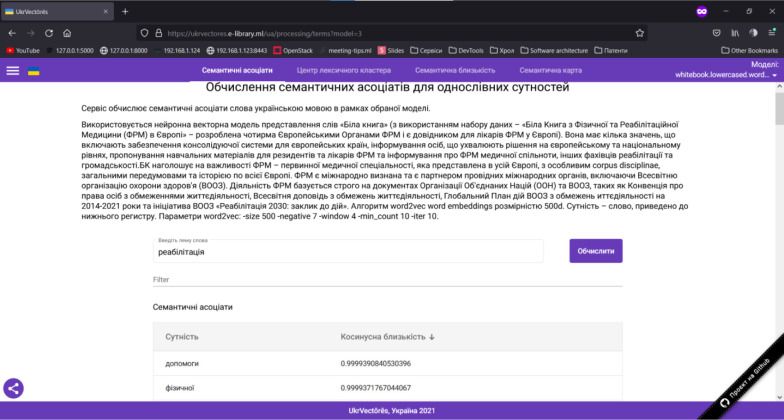
GUI of the UkrVectōrēs SPA Service (“Semantic Associates” Mode, the Select Component Called “Models”)

**Figure 4:**
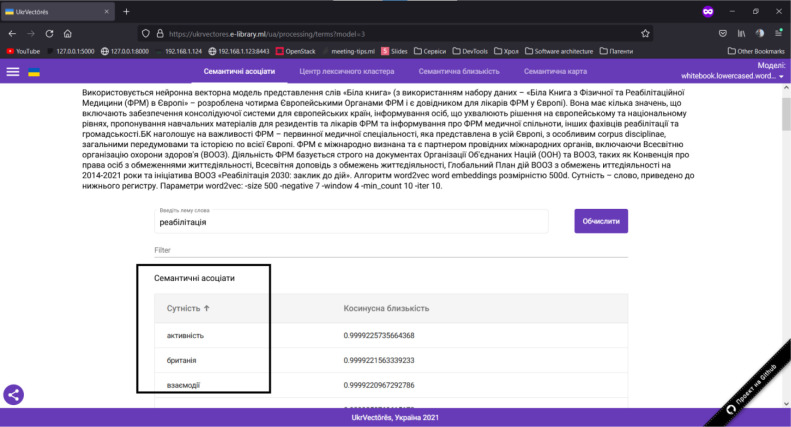
GUI of the UkrVectōrēs SPA Service (“Semantic Associates” Mode, the “Entity” Element)

**Figure 5:**
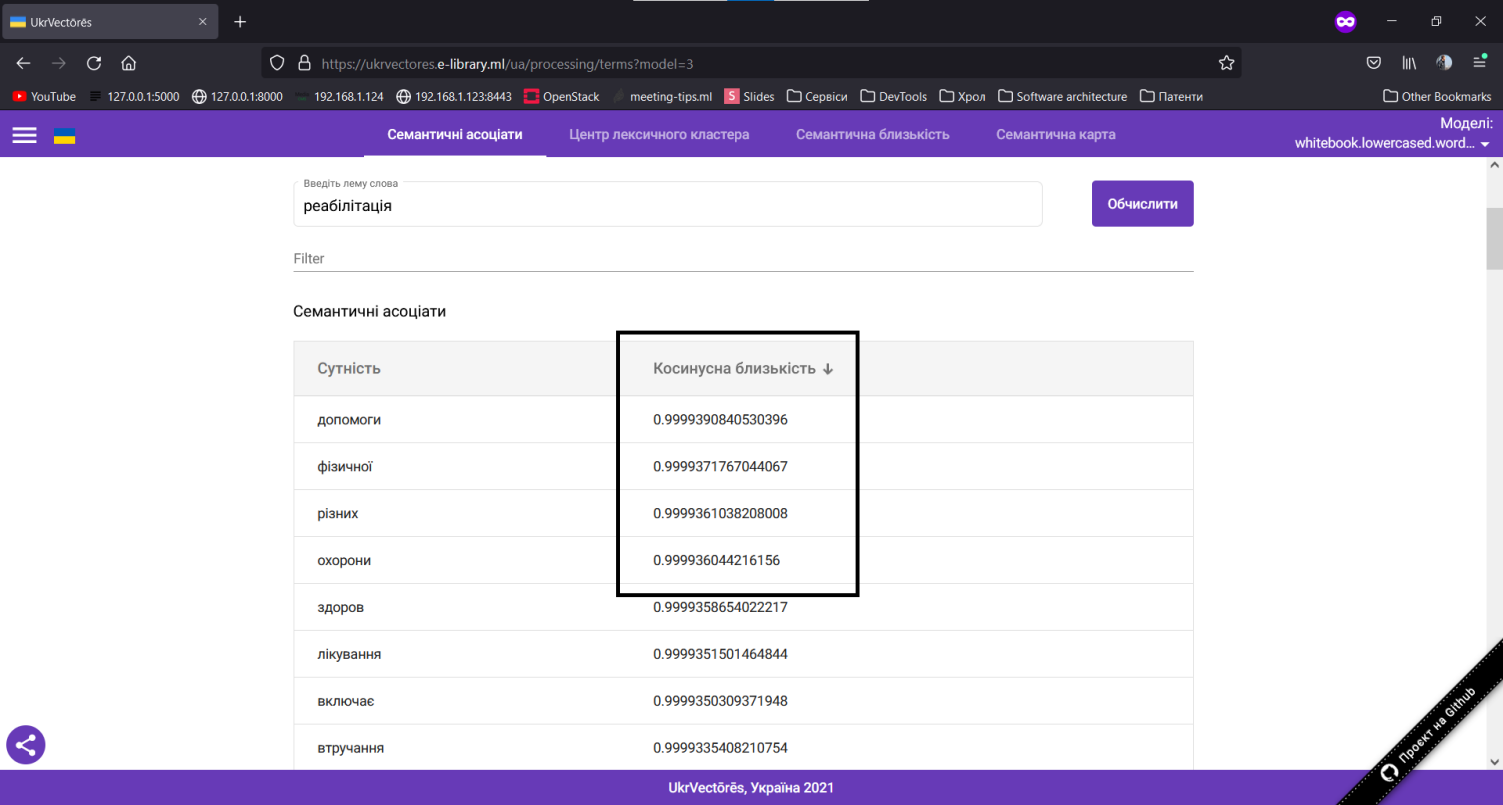
GUI of the UkrVectōrēs SPA Service (“Semantic Associates” Mode, the “Cosine Similarity” Element)

*Computation of the semantic associates for a given entity (word)* within the selected distribution-semantic model. To use this function, you need to:

1. Open the graphical user interface (GUI) of the UkrVectōrēs Single Page Application (SPA) using the current version of Google Chrome, Mozilla Firefox, or Microsoft Edge web browser. To do this, enter the following link in the address bar of the web browser: https://ukrvectores.ai-service.ml/ (the link may differ, it depends on the deployment features of the UkrVectōrēs service) and select the Semantic Associates operation mode in the main menu, as shown in Figure 2.
2. Using the drop-down list of the select component named Models (in Ukrainian – Моделі) in Figure 3, choose the desired distributional semantic model, within which the computation of semantic associates will be carried out (by default the neural vector model for words representation “White Book” is used (using the “White Paper on Physical and Rehabilitation Medicine in Europe” dataset), word2vec word embeddings algorithm with the dimension of 500d. The entity is a word, lemmatized, reduced to lower case. Hyperparameters of word2vec are: *-size 500 -negative 5 -window 5 -threads 24 -min_count 10 -iter 20*).
3. In the field of the input component named “Enter the word lemma,” specify the desired word lemma for which you want to compute semantic associates (for example, rehabilitation (in Ukrainian – реабілітація), as shown in Figure 2) and press the “Enter” key or the “Compute” button.
4. Semantic Associates will be displayed on the screen as shown in Figure 2 (by default, the first 100 associates for decreasing the cosine similarity coefficient are displayed) for the given lemma of the word rehabilitation (in Ukrainian – реабілітація) within the selected distributional semantic model “White Book”.
5. Using the “Entity” (in Ukrainian – “Сутність”) element, the user can choose to display the semantic associates alphabetically as shown in Figure 4.
6. Using the “Cosine similarity” (in Ukrainian – “Косинусна близькість”) element, the user can choose to display the semantic associates by the cosine similarity coefficient (by increasing or decreasing) as shown in Figure 5.

**Figure 6:**
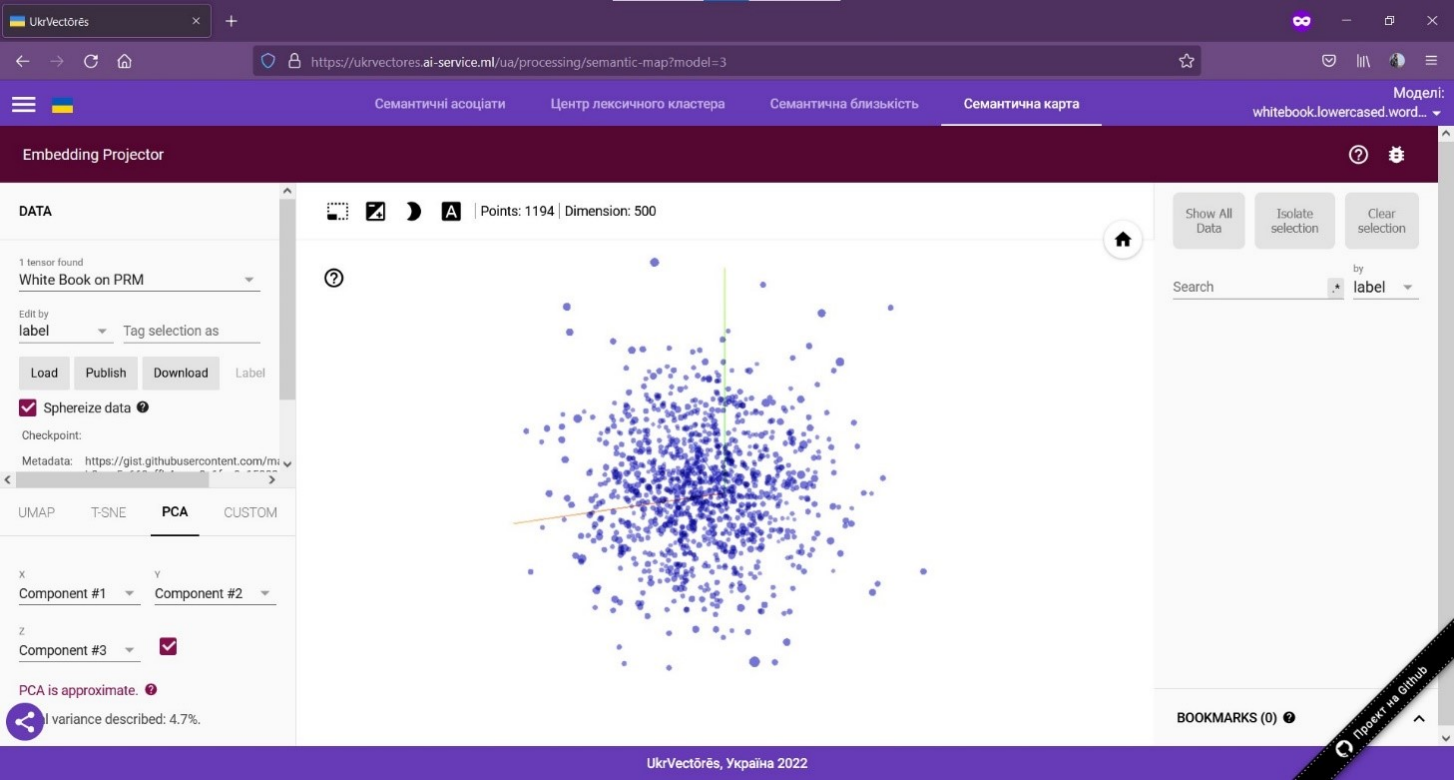
GUI of the UkrVectōrēs SPA Service (“Semantic Map” Mode)

**Figure 7:**
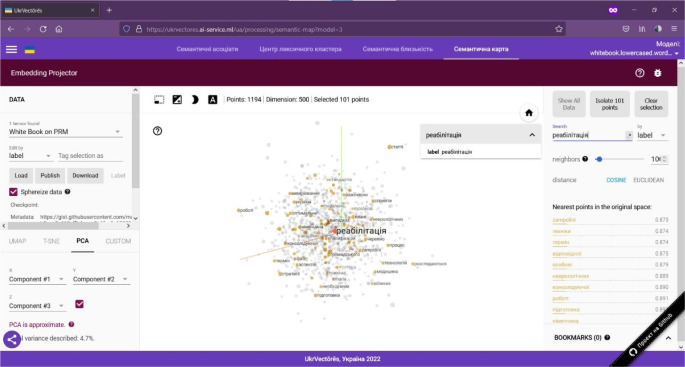
Visualization of the Semantic Associates of the Lemma of the Word in Ukrainian – реабілітація

Generation of semantic maps (using the open-source software toolkit TensorFlow, namely TensorBoard) of relations between words within the selected distributional semantic model. To use this function, you need to:

1. Open the GUI of the UkrVectōrēs SPA using the current version of Google Chrome, Mozilla Firefox, or Microsoft Edge web browser. To do this, enter the following link in the address bar of the web browser: https://ukrvectores.ai-service.ml (the link may differ, it depends on the deployment features of the UkrVectōrēs service) and select the Semantic map mode in the main menu, as shown in Figure 6.
2. Using the drop-down list of the select component named Models (in Ukrainian – Моделі) in Figure 6, choose the desired distributional semantic model, within which the computation of semantic associates will be carried out (by default the neural vector model for words representation “White Book” is used (using the “White Paper on Physical and Rehabilitation Medicine in Europe” dataset), word2vec word embeddings algorithm with the dimension of 500d. The entity is a word, lemmatized, reduced to lower case. Hyperparameters of word2vec are: - size 500 -negative 5 -window 5 -threads 24 -min_count 10 -iter 20).
3. An example of visualization of the semantic associates of the lemma of the word “rehabilitation” (in Ukrainian – реабілітація) is shown in Figure 7.

The compilation & deployment technologies, and more detailed description of the source code of the UkrVectōrēs service, as well as the methodology for training the distributional semantic model of the vector representation of entities (using the dataset - “White Paper on Physical and Rehabilitation Medicine (PRM) in Europe”), are available in (UkrVectōrēs, n.d.). Currently, the most recent version of the UkrVectōrēs service is available at https://ukrvectores.ai-service.ml/ and is free for use in R&D and teaching purposes.

## VHEALTH – THE DIGITAL LIBRARY OF MEDIA CONTENT

The digital library of media content of the TISP Smart-system subsystem - the vHealth service (vHealth, n.d.) is a distributed information system that allows the storage, use, and sharing of various collections of electronic documents (video and audio content) of arbitrary domain areas, for distance learning of patients and their relatives, in particular, a rehabilitation set of exercises.

One of the main tasks and purposes of the vHealth service is the integration of information resources and efficient navigation within them. Integration of information resources is their unification to use different information while preserving its properties, presentation features, and the ability to process it. The pooling of resources can occur both physically and virtually. However, at the same time, such a combination should provide the user with the perception of the necessary information as a single information space: an electronic library should ensure work with databases and high efficiency of the information search. Effective navigation in the electronic library is the ability of the user to find the information of interest to them in all available information space with the greatest completeness and accuracy at the least effort. To solve this problem, the vHealth service uses smart search based on predictive models of distributional semantics.

The vHealth service has the following features:

- complete control over media content and user data;
- support for multiple publishing workflows: public, private, unlisted, and custom;
- types of multiple media support: video, audio, image, pdf (and docx in future releases);
- multiple media classification options: categories, tags, and custom;
- intelligent information search in real time based on the predictive models of distributional semantics (lexical, symbolic, and attribute search);
- playlists for audio and video content: create playlists, add, and reorder content;
- SPA responsive design: including light and dark themes;
- advanced users' management: allows self-registration, invite only, closed;
- configurable actions: allows download, add comments, add likes, dislikes, report media;
- enhanced video player: customized video.js player with multiple resolution and playback speed options;
- multiple transcoding profiles: sane defaults for multiple dimensions (240p, 360p, 480p, 720p, 1080p) and multiple profiles (h264, h265, vp9);
- chunked file uploads: for pausable/resumable upload of content;
- logging of the user's session with the system with the ability to switch to each of the previously existing system states;
- manipulating the structure of the description of the media content object;
- support of the appliance of hypertext and hypermedia links, which provides the user with a quick transition from an object or its element to another interrelated object or element.

*Let us consider a brief look of the user experience with the graphical user interface of the vHealth single-page application*.

The GUI of the popular media platform Youtube, which is the standard for media content distribution systems, was taken as the basis for the development of the GUI of the vHealth SPA. During the development of the GUI of the vHealth network tool, one of the most important requirements for the modern graphical interface and user experience of the software system was met - the Do What I Mean concept. Therefore, the system works predictably, so that the user intuitively understands in advance what action the program will perform after receiving his command. This greatly improves the interaction of the user with the system and does not require the development of additional techniques and user settings for interacting with the graphical interface of the software system.

**Figure 8:**
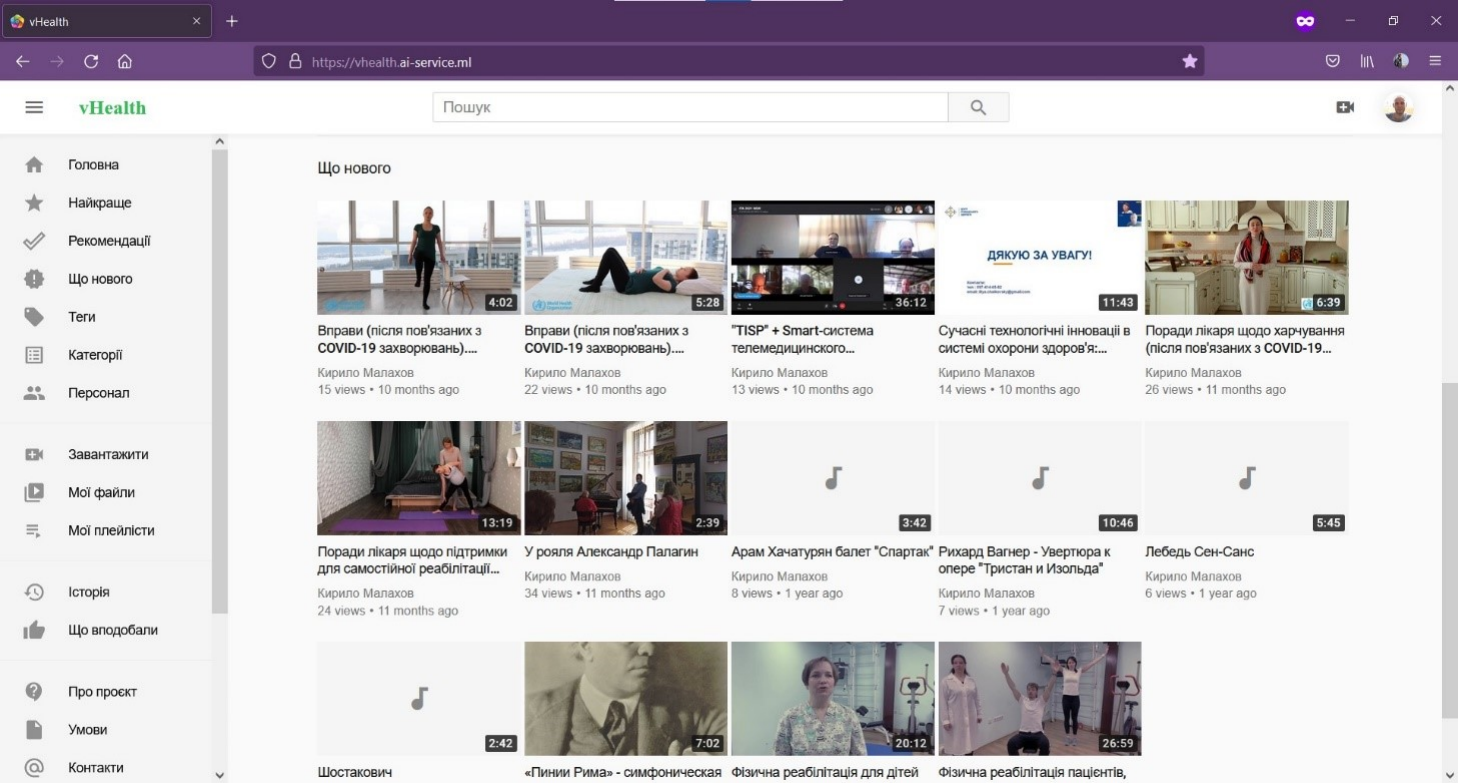
GUI of the vHealth SPA Service (Home Page)

The main elements of the GUI of the vHealth SPA are shown in Figure 8:

- the central part of the interface contains all media content available to the user according to his profile. Media content (by default) is categorized;
- the part of the interface on the left side contains the items of the main menu of the vHealth network tool, in particular, the items: “Upload” (in Ukrainian – Завантажити) (the function of uploading new media content); “My files” (in Ukrainian – Мої файли) (the function of viewing the uploaded media content in the user profile); “My playlists” (in Ukrainian – Мої плейлісти) (formation of playlists - playlists of media content with setting up workflows for publishing content); “History” (in Ukrainian – Історія) (function of viewing a list of media content that has already been viewed); Stuff (in Ukrainian – Персонал) (function of viewing all users of the vHealth service); “Categories” and “Tags” (the function of cataloging media content objects and their various associations);
- the graphical interface of the user's profile (account) is shown in Figure 9. The user has access to personal media content and has the ability to edit the graphical interface of his profile and each media content object. There is also access to the user's playlists page.

**Figure 9:** GUI of the vHealth SPA Service (User Profile Page) *Editor's Comment*: Figure 9 is identical to Figure 8 except that a user photo and name appear at the top of the screen shot. Therefore, the figure has been omitted to preserve user privacy.

The compilation, deployment, and a more detailed description of the source code of the vHealth service, as well as the methodology of user interaction with the GUI of the vHealth application, require a separate review and is beyond the scope of this article. Currently, the most recent version of the vHealth service is available at https://vhealth.ai-service.ml/ and is free for use f R&D and teaching purposes. To start working with the vHealth service, you need to be an authorized user (log in), so the demo account was created to demonstrate how the service works. You can log in using the login and password of the demo profile account (*login: demouser; password: JyMyuC6nMdD494T*).

## DISCUSSION

The top-priority challenges were faced by the medical rehabilitation system in Ukraine. Particularly important tasks include, first of all, the rehabilitation of patients who have recovered from COVID-19 disease. This fact is well understood both by the society and the leadership of the Ministry of Health of Ukraine, which is creating a special working group on this problem. Ukraine has a system of medical and prophylactic institutions designed for psychological and physical rehabilitation of military personnel; these use modern rehabilitation technologies. However, long-term rehabilitation in such centers is not available to everyone. Therefore, the use of telerehabilitation technology for patients with post-traumatic stress disorder and similar disorders in combination with a means of objective control of the functional state is extremely important.

In a large-scale pandemic, the number of patients undergoing rehabilitation therapy is growing unexpectedly. Therefore, it is very important today to create intelligent information-analytical systems to support rehabilitation processes, which solve a set of problems related to improving the effectiveness of combating such a global natural phenomenon.

The V.M. Glushkov Institute of Cybernetics of the National Academy of Sciences of Ukraine (InCyb, n.d.) is currently working on a project of the National Research Foundation of Ukraine (NRFU, n.d.) entitled “Development of the cloud-based platform for patient-centered telerehabilitation of oncology patients with mathematical-related modeling.”

A special role in the implementation of all functionalities for the rehabilitation of patients in a large-scale pandemic includes:

- support of information-analytical processes of data collection and processing;
- classification of patients by health status;
- methodological recommendations for comprehensive rehabilitation;
- study of the fundamental causes and consequences of crisis (see pandemic) situations in the country and in the world;
- support for decision-making at all levels of public administration, development, and correction of the general policy of development of the state and its separate regions;
- providing information and advisory assistance to the population;
- methodological principles of recovery and rehabilitation in accordance with the recommendations of the WB and ICF.

The project is based on cognitive information technology, which fully provides the process of analyzing large amounts of information resources. Cognitive services implement structuring and classification of information, synthesize the necessary documents based on semantic analysis, identify the characteristic properties of information processes and provide decision support at all stages of their life cycle: assessment of the patient's condition, formulation of rehabilitation diagnosis and rehabilitation goal, rehabilitation program assessment of functional consequences, determination of rehabilitation potential taking into account the nature of diseases, patient resources, as well as psychological and socio-environmental factors indicated by the ICF, adopted by the World Health Organization (WHO).

The peculiarity of the system is that it is based on knowledge-oriented technology, ontology engineering, and a transdisciplinary paradigm.

Rehabilitation specialists are faced with the need to determine the patient's rehabilitation potential for further routing and prognosis of recovery, as well as ongoing monitoring of the effectiveness of rehabilitation interventions. On the basis of this assessment, a rehabilitation plan and program are developed, indicating the prognosis for achieving certain tasks. There are numerous scales of functional assessment to assess rehabilitation potential. However, the analysis of these scales reveals the absence of specialized scales for assessing the cardiorespiratory system.

In recent years, the Institute of Cybernetics team has developed an innovative method of scaling the electrocardiogram and heart rate variability. We have developed a method (Universal Scoring System) and software for scaling the electrocardiogram, which is able to quantify the smallest changes in the electrocardiographic signal. The idea of our approach is first to measure the maximum number of routine and original electrocardiogram ECG parameters and heart rate variability, and second, to position each parameter on the scale between the absolute norm and extreme pathology.

This technique is successfully used for functional assessment in the process of telerehabilitation of patients recovering after COVID-19 as well as patients with post-stress disorders.

## CONCLUSION

The purpose of our research was to develop a formal model, software implementation, and the methodological foundations for the use of services of the remote Patient / Personal-centered Smart-system for providing medical rehabilitation assistance to patients in a pandemic.

In this paper, we introduced and defined:

- the basic concepts of the new Hybrid E-rehabilitation notion and its fundamental foundations;
- the formalization concept of the new Patient / Person-centered Smart-system for remote support of rehabilitation activities and services;
- the methodological foundations for the use of services (UkrVectōrēs and vHealth) of the remote Patient / Person-centered Smart-system.

The software implementation of the services of the Smart-system for remote support of rehabilitation activities and services has been developed, in particular:

- the digital library of media content of the telerehabilitation subsystem of TISP – the vHealth service;
- the UkrVectōrēs service - NLU-powered network tool for knowledge discovery, classification, diagnostics, and prediction.

The research results were presented by our team on the All-Ukrainian forum “Ukraine 30: Education and Science” in section - “Big data Ukraine platform.” Also, the research results (in teleconference and offline) were presented during the workshop “Digital services and devices for rehabilitation measures support.”

This study can be extended by future research in several directions, both in theory and in practice. Further research is also planned in the definition and application of efficient mathematical methods for big data analysis; modeling and creating scenarios (workflows) for predicting and optimizing the entire complex of rehabilitation procedures and their routing using system tools; and technologies, and experience in the development of rehabilitation systems and complexes that have already been tested by our team.

## FURTHER RESEARCH

In a future study, our teams plan to implement HIS to optimize the time spent by specialists of the multidisciplinary team in the use of ICF in the rehabilitation of cancer patients (including breast cancer). Further research will aim to determine and apply effective mathematical methods of big data analysis, modeling and developing of prediction scenarios, and optimization of the full set of rehabilitation procedures and their routing. This will employ tools already tested in the team system, technologies, and experience in developing rehabilitation systems.
